# LncRNA RSU1P2-microRNA let-7a-Testis-Expressed Protein 10 axis modulates tumorigenesis and cancer stem cell-like properties in liver cancer

**DOI:** 10.1080/21655979.2022.2031394

**Published:** 2022-02-14

**Authors:** Jia-Hong Liang, Qiao-Dong Xu, Song-Gang Gu

**Affiliations:** a Department of Biliary-Pancreatic Minimally Invasive Surgery, The First Affiliated Hospital of Shantou University Medical College, Shantou, China; b Department of Hepatobiliary surgery, Cancer Hospital of Shantou University Medical College, Shantou, China

**Keywords:** LncRNA RSU1P2, let-7a, Tex10, tumorigenesis, cancer stem cell-like properties, liver cancer

## Abstract

LncRNAs exert important functions in the modulation of tumorigenesis and cancer stem cell-like properties in liver cancer. However, the role of LncRNA Ras suppressor protein 1 pseudogene 2 (RSU1P2) in modulating tumorigenesis and cancer stem cell-like properties in liver cancer is still not known. In this study, the expression of LncRNA RSU1P2 was significantly elevated in liver cancer tissues and cells. Besides, knockdown of RSU1P2 repressed cell viability, invasion, epithelial-mesenchymal transition (EMT) of liver cancer cells and the expressions of cancer stem cell-related genes, whereas facilitated the apoptosis of liver cancer cells. In addition, LncRNA RSU1P2 can interact with microRNA let-7a (let-7a), and repress let-7a expression. Testis-Expressed Protein 10 (Tex10) was identified to be a target of let-7a, and let-7a repressed Tex10 expression. Finally, RSU1P2 knockdown suppressed tumor volume, tumor weight, and EMT in a xenograft model. Therefore, LncRNA RSU1P2 promotes tumorigenesis and cancer stem cell-like properties in liver cancer through let-7a/Tex10 pathway.

## Introduction

Liver cancer is the fourth most common cancer deaths in digestive system in the United States [1]. It has been estimated that new cases will reach to 42,810 and the deaths will reach to 30,160 in the United States according to the 2020 Cancer Statistics [2]. In China, liver cancer is the second cause of cancer death in male and the third in female, and liver cancer is mainly caused by nonalcoholic steatohepatitis, hepatitis C virus and hepatitis B virus [3]. The progression of liver cancer is complex which is related with liver cancer cells proliferation, migration and invasion and cancer stem cell-like properties [4,5]. According to previous reports, cancer stem cells are in charge of liver cancer growth and metastasis, as well as related with the recurrence of liver cancer [6,7]. Although many high or low expressed genes involved in tumorigenesis and cancer stem cell-like properties of liver cancer were identified, the underlying molecular mechanisms for liver cancer are not fully understood [5,8]. Therefore, underlying mechanisms that regulate liver cancer tumorigenesis and cancer stem cell-like properties should be identified to develop efficient strategies for the treatment of liver cancer.

Many studies have found that a variety of long non-coding RNAs (LncRNAs), such as LncRNA plasmacytoma variant translocation 1 (PVT1), LncRNA pituitary tumor-transforming 3, pseudogene (PTTG3P) and LncRNA n335586, can promote the proliferation, migration, invasion and cancer stem cell-like properties in liver cancer [9–11]. LncRNA Ras suppressor protein 1 pseudogene 2 (RSU1P2) is a newly discovered LncRNA that acts as a cancer-promoting gene in cervical cancer, and LncRNA RSU1P2 can act as a competitive endogenous RNA (ceRNA) against let-7a to promote its downstream molecules insulin-like growth factor 1 receptor (IGF1R), N-myc and EphA4 expressions, thereby promoting tumorigenesis and epithelial-mesenchymal transition (EMT) in cervical cancer [12]. However, the effect of LncRNA RSU1P2 in liver cancer progression is still unrevealed.

Let-7a is a disease-associated microRNA (miRNA) that can modulate biological functions involving the proliferation, migration, and invasion, EMT and cancer stem cell-like properties in cancers, including lung cancer, nasopharyngeal carcinoma, and breast cancer [13–15]. According to previous reports, let-7a is considered as being reduced in the progression of cancers, and let-7a can modulate the oncogenes expressions and facilitate colon cancer and pancreatic cancer tumorigenesis [16,17]. In addition, researchers have shown that let-7a can suppress the sphere formation efficiency of liver cancer cells, EMT, renewal of cancer stem cells and Wnt-β-catenin pathway in liver cancer [18]. EMT can contribute to the invasion and metastasis of liver cancer [19,20]. Therefore, let-7a might exert a vital function in modulating tumorigenesis and cancer stem cell-like properties in liver cancer.

Testis-Expressed Protein 10 (Tex10) was firstly reported to regulate ribosome biogenesis in eukaryotes [21]. Later, Tex10 has been shown to link arginine methylation to desumoylation and modulate transcriptional activity [22]. In addition, Tex10, as an important pluripotency factor, exerts vital function in self-renewal of embryonic stem cells, pluripotency maintenance, embryo development, and reprogramming [23,24]. Importantly, Tex10 has been found to be elevated in liver cancer cells and liver cancer stem cells, as well as Tex10 overexpression facilitates cancer stem cell properties by activating STAT3 pathway [25].

This study assumes that LncRNA RSU1P2 is elevated in liver cancer tissues and cells, and acts as a cancer-promoting gene in liver cancer. We aim to investigate the promoting role of LncRNA RSU1P2 in liver cancer cells proliferation, invasion, EMT, and cancer stem cell-like properties, and its underlying mechanism, which might provide potential targets for liver cancer treatment.

## Materials and methods

### Sample selection

Liver cancer tissues and tumor adjacent tissues (35 pairs) were obtained from liver cancer patients (25 male and 10 female patients; aged from 45 to 69 years old) when they underwent hepatectomy at The First Affiliated Hospital of Shantou University Medical College from January 2019 to October 2019, which were confirmed by two histopathologists. Informed consent was obtained from all patients. All liver cancer patients did not receive pre-operative treatments, such as immunotherapy, targeted therapy, and chemotherapy [26]. All liver cancer tissues and tumor adjacent tissues were stored at −80°C. This experiment was approved by the Ethics Committee of The First Affiliated Hospital of Shantou University Medical College.

### Cell culture and transfection

Liver cancer cell lines HepG2, HCCLM3, Huh7, and Hep3B, and normal liver cell-line THLE-2 were obtained from the Cell Bank of Chinese Academy of Sciences (Shanghai, China) or maintained in our laboratory. Cells were incubated in Minimum Essential Media (MEM; Gibco, CA, USA) with 10% fetal bovine serum (FBS; Gibco), 1% glutamax (Gibco), 1% non-essential amino acids (Gibco), 1% sodium pyruvate (Sinopharm Chemical Reagent Co., Ltd, Shanghai, China), 100 μg/mL streptomycin and 100 U/mL penicillin (Invitrogen, CA, USA) in a 5% CO_2_ incubator at 37°C [27].

Small interfering RNA (siRNA) targeting RSU1P2 (si-RSU1P2-1 and si-RSU1P2-2) and siRNA negative control (si-NC) were synthesized by GENECHEM (Shanghai, China). Let-7a mimic and negative control mimic (mimic-NC) were obtained from RiboBio Co., Ltd. (Guangzhou, China). The plasmids pcDNA-RSU1P2 and pcDNA-Tex10 were generated by cloning their fragments, which were amplified by their special primers, into the pcDNA3.1 vector, respectively. The sequences of the siRNAs, let-7a mimic, mimic-NC, and the primers are shown in Table 1. HCCLM3 and HepG2 cells were transfected with these plasmids using Lipofectamine reagent (Invitrogen, CA, USA).

**Table 1.** Sequences of the oligonucleotides

Name	Sequence (5’-3’)
si-RSU1P2-1	UUACUAUGUGAUUUGUACCUG
si-RSU1P2-2	UUAACCUCUAUUCUCUACUGU
si-NC	CUACACCGUAUUCUACUACUA
Let-7a mimic	UGAGGUAGUAGGUUGUAUAGUU
Mimic-NC	UUCUCCGAACGUGUCACGUTT
RSU1P2- forward primers	CGGGATCCACTTGAATAACCAAACCTATGCC
RSU1P2- reverse primers	CCGGAATTCGAGAATTACTATGTGATTTGTACC
Tex10- forward primers	CCGAAGCTTATGACTAAAAAAAGAAAACGCCAAC
Tex10- reverse primers	TGCTCTAGATCAATACACTGTAGCCAGTGCACTG

### Reverse transcription-quantitative polymerase chain reaction (qRT-PCR)

TRIzol reagent (Invitrogen, CA, USA) was conducted to isolate total RNAs from liver cancer tissues and cells. After that, RNAs were reversely transcribed into cDNA by RNA to cDNA EcoDry Premix (Clontech, Dalian, China). Then, qRT-PCR was performed by One Step TB Green PrimeScript RT-PCR Kit (Takara, Dalian, China) on a Thermal Cycler Dice Real-Time System II (Takara, Dalian, China). The relative gene expressions of RSU1P2, let-7a, Tex10, ATP-binding cassette (ABC) superfamily G member 2 (ABCG2), NANOG, aldehyde dehydrogenase 1 (ALDH1), octamer-binding transcription factor 4 (OCT4) and SOX2 were calculated to that of the 2^−ΔΔCt^ method [28] and normalized by GAPDH or U6. Primers for amplification are shown in Table 2.

**Table 2.** Sequences of primers used for qRT-PCR

Name	Forward primers (5’-3’)	Reverse primers (5’-3’)
RSU1P2	GGACATCGAGAAACTAAAG	GGTCACAGAACAAGGGAG
let-7a	GGAGAATTCGAAACCAGGATTACCGAGG	GGAGACTCGAGCAAATGCTGCACTACATCTC
Tex10	TTGCAACTTGCTCATCTTGG	AGAGTCTGCAGGGAGAACCA
ABCG2	ACAACCATTGCATCTTGTCTGTC	GCTGCAAAGCCGTAAATCCATATC
NANOG	AATACCTCAGCCTCCAGCAGATG	TGCGTCACACCATTGCTATTCTTC
ALDH1	TCCAGCCCACAGTGTTCTCTAAT	GATTTGCTGCACTGGTCCAA
OCT4	CTTGCTGCAGAAGTGGGTGGAGGAA	CTGCAGTGTGGGTTTCGGGCA
SOX2	CGCAGACCTACATGAACG	CCCTGGAGTGGGAGGAA
GAPDH	TGCACCACCAACTGCTTAGC	GGCATGGACTGTGGTCATGAG
U6	TGCGGGTGCTCGCTTCGGCAGC	CCAGTGCAGGGTCCGAGGT

### Viability MTT (3-(4,5-dimethylthiazol-2-yl)-2,5-diphenyltetrazolium bromide) assay

MTT Assay Kit (Beyotime Biotechnology, Nantong, China) was used to measure the effects of RSU1P2, let-7a and Tex10 on cell viability of HCCLM3 and HepG2 cells. HCCLM3 or HepG2 cells (2 × 10^3^/well) were cultured in 96-well plates for 48 h [29]. MTT solution (10 μL) was added into each well, and the plates were cultured for 4 h at 25°C. After that, formazan solution (100 μL/well) was added, and the plates were cultured for 3 h at 37°C. Finally, absorbance at 570 nm was measured by a microplate reader (Bio-Rad, CA, USA).

### Colony formation assay

Since liver cancer cells can proliferate in suspension, the malignant proliferation ability can be reflected by the ability of colony formation in plates [30]. After transfection for 48 h, HCCLM3 and HepG2 cells were counted and cells (2 × 10^3^/well) were put into each well of 6-well plates with DMEM (Gibco) for colony formation assay for 2 weeks. The colonies of HCCLM3 and HepG2 cells (approximately 50 or more cells) were fixed with 4% paraformaldehyde (Sinopharm Chemical Reagent Co., Ltd, Shanghai, China) for half an hour. Finally, the colonies of HCCLM3 and HepG2 cells were stained with crystal violet (0.5%; Sinopharm Chemical Reagent Co., Ltd, Shanghai, China) for half an hour at 25°C. The colonies of HCCLM3 and HepG2 cells were observed with a light microscope (Olympus, Tokyo, Japan).

### Apoptosis assay

HCCLM3 and HepG2 cells at different groups were harvested with trypsin, washed with cold phosphate buffer solution (PBS), and immobilized with 70% ethanol at 4°C overnight. HCCLM3 and HepG2 cells (1 × 10^6^ cells) were then re-suspended in Annexin V-FITC binding buffer (195 μL; Beyotime Biotechnology, Nantong, China), followed by the addition of Annexin V-FITC (5 μL; Beyotime Biotechnology, Nantong, China), and stained with 10 μL propidium iodide at 25°C in the dark for 20 min. FACS analysis was conducted with a flow cytometer (FACScan; BD, NJ, USA), and data were analyzed by Cell Quest software. Cell apoptosis (%) = number of total apoptotic cells (early+late apoptosis)/number of total cells × 100% [31].

### Transwell cell invasion assay

For the observation of cell invasion ability, 0.2 ml HCCLM3 and HepG2 cells (1 × 10^5^ cells) were added in the upper chambers of 8 μM pore-sized Transwell (Corning, NY, USA) which were pre-coated with 1:8 matrigel. HCCLM3 and HepG2 cells were incubated in Transwell for 24 h. Then, HCCLM3 and HepG2 cells remained in the upper chambers were removed. Cells invaded to lower chambers were immobilized with 4% paraformaldehyde (Sinopharm Chemical Reagent Co., Ltd, Shanghai, China) and then stained with crystal violet (0.3%; Beyotime Biotechnology, Nantong, China). Invasive cells were counted by a light microscope (Olympus, Tokyo, Japan) [32].

### Western blotting for the detection of proteins

Tumor tissues and HCCLM3 and HepG2 cells were lysed using RIPA buffer (ThermoFisher Scientific, CA, USA) to obtain total proteins. Proteins were separated by SDS-PAGE and transferred to PVDF membranes. The above PVDF membranes were incubated with corresponding primary antibodies at 4°C overnight: anti-E-cadherin (1:1000; Cell Signaling Technology, MA, USA), anti-Vimentin (1:500; Sigma-Aldrich, MO, USA), anti-Tex10 (1:500; Proteintech Biotechnology, IL, USA), anti-Wnt1 (1:1000; Abcam, Cambridge, UK), anti-β-catenin (1:5000; Abcam), and anti-GAPDH (1:5000; Abcam). Then, the membranes were incubated with HRP-conjugated secondary antibody at 25°C for 2 h. Detection was conducted by enhanced chemiluminescence kit (ECL; ThermoFisher Scientific, CA, USA) [33].

### Dual luciferase reporter gene assay

The interaction between let-7a and Tex10 was predicted by StarBase (http://starbase.sysu.edu.cn/). We firstly selected a species, then enter a gene symbol, and clicked on submit button. DIANATools (http://diana.imis.athena-innovation.gr/DianaTools/index.php) was used to predict the binding sites between RSU1P2 and let-7a. We firstly clicked on SOFTWARE button, chose LncBase v.2, then selected prediction module. The sequence of RSU1P2 or 3’-untranslated regions (3’-UTR) of Tex10 was sub-cloned into pGL3 luciferase reporter vectors (Promega, WI, USA), respectively. Quick-change site-directed mutagenesis kit (Agilent Technologies, CA, USA) was used to mutate the potential has-let-7a binding sites. The wide type 3’-UTR of Tex10 vector (pGL3-Tex10 wt), mutant 3’-UTR of Tex10 vector (pGL3-Tex10 mut), wide-type RSU1P2 vector (pGL3-RSU1P2 wt), mutant RSU1P2 vector (pGL3-RSU1P2 mut), mimic-negative control (mimic-NC) or let-7a mimic were co-transfected into HepG2 and HCCLM3 cells. Dual Luciferase Reporter Assay System (Promega, WI, USA) was conducted to examine the luciferase activity at 48 h [34].

### Xenograft model for assess the effect of RSU1P2 on tumor growth and EMT

Male BALB/c nude mice (4–5 weeks, weighing 18–20 g) were kept in a 12 h light/12 h dark condition with free access to water and food. Nude mice were randomly divided into sh-NC and sh-RSU1P2 groups, with five mice in each group. To establish xenograft tumor model, HCCLM3 cells were transfected with sh-NC and sh-RSU1P2 (GenePharma, Shanghai, China). Briefly, HCCLM3 cells (1 × 10^5^) were put to each well of 24-well plates and cultured for 24 h. sh-NC or sh-RSU1P2 (1 μg) was added into cells and incubated for 72 h. Then, HCCLM3 cells (1 × 10^6^ cells, 200 μL) were injected into the right flank of nude mice subcutaneously [35]. Tumor volume was recorded every six days and calculated by the formula: volume = (length × width^2^)/2. Mice were euthanized by an overdose of 100 mg/kg sodium pentobarbital through intravenous injection. Tumor tissues were collected from sacrificed mice at day 30. This animal experiment was approved by the Animal Care and Use Committee of The First Affiliated Hospital of Shantou University Medical College (SYXK(Yue)2019–0079).

### Immunohistochemical (IHC) staining

Tumor tissue sections were treated with 1% H_2_O_2_, blocked with 1% FBS (Gibco, CA, USA), and immunostained with anti-E-cadherin (1:1000; Cell Signaling Technology, MA, USA) or anti-Vimentin (1:500; Sigma-Aldrich, MO, USA) for 12 h at 4°C. After that, tumor tissue sections were incubated with a secondary antibody (1:500; Cell Signaling Technology) for 2 h at 25°C. All sections were developed by 3,3’-diaminobenzidine (DAB) kit (Thermo Scientific, CA, USA) [36].

### Statistical analysis

SPSS 18.0 (Chicago, USA) was performed for data analysis, and data were presented as mean ± standard deviation. One-way analysis of variance (ANOVA) followed by Bonferroni post hoc test was used to analyze differences among multiple groups. Student’s t-test was used to analyze differences between two groups. Significance levels were set at p value less than 0.05 [37].

## Results

We aim to investigate the promoting role of LncRNA RSU1P2 in liver cancer and the underlying mechanism of LncRNA RSU1P2 in liver cancer cells proliferation, invasion, EMT and cancer stem cell-like properties *in vitro* and tumorigenesis *in vivo*. We proved that let-7a expression was regulated by LncRNA RSU1P2, and let-7a could target to Tex10. We verified that LncRNA RSU1P2/let-7a/Tex10 pathway promoted liver cancer cell proliferation, invasion, EMT and the expression of cancer stem cell-related genes *in vitro* and confirmed our hypothesis *in vivo*.

### LncRNA RSU1P2 was elevated in cancer tissues from liver cancer patients and liver cancer cell lines

**Figure 1.**
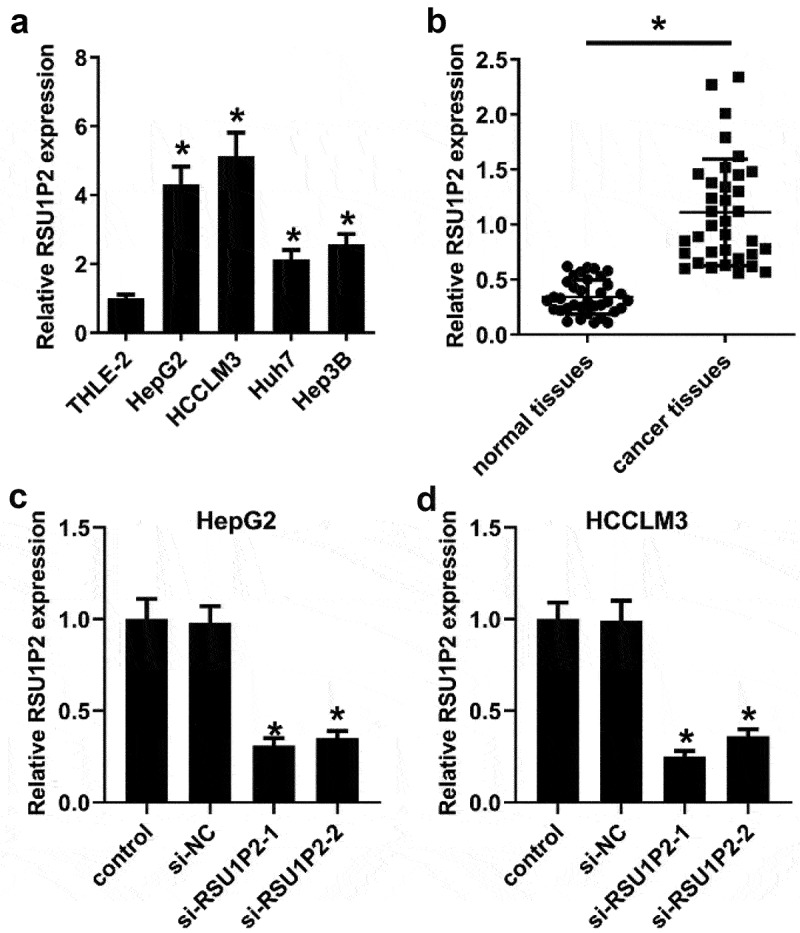
LncRNA RSU1P2 was raised in cancer tissues from liver cancer patients and liver cancer cell lines. (a). LncRNA RSU1P2 expression in different liver cancer cell lines was examined using qRT-PCR. *p < 0.05, compared with THLE-2 group. (b). LncRNA RSU1P2 expression in normal tissues and cancer tissues was measured using qRT-PCR. *p < 0.05, compared with normal tissue group. C-D. HCCLM3 and HepG2 cells were transfected with siRNA negative control (si-NC), si-RSU1P2-1 or si-RSU1P2-2. LncRNA RSU1P2 expression in HCCLM3 and HepG2 cells was measured using qRT-PCR. *p < 0.05, compared with si-NC group.

The expression of LncRNA RSU1P2 was significantly elevated in liver cancer cell lines HepG2, HCCLM3, Huh7, and Hep3B than that in normal liver cell-line THLE-2, especially in HepG2 and HCCLM3 cells (1.00 ± 0.11 vs 4.32 ± 0.51, 1.00 ± 0.11 vs 5.14 ± 0.68, p < 0.05; Figure 1(a)), which was also significantly up-regulated in liver cancer tissues compared to normal tissues (0.34 ± 0.16 vs 1.11 ± 0.48, p < 0.05; Figure 1(b)). After knockdown of RSU1P2 (si-RSU1P2-1, si-RSU1P2-2), LncRNA RSU1P2 expression was significantly repressed in HCCLM3 and HepG2 cells than that in control group (HepG2 cells: 1.00 ± 0.11 vs 0.31 ± 0.04, 1.00 ± 0.11 vs 0.35 ± 0.04, p < 0.05; HCCLM3 cells: 1.00 ± 0.09 vs 0.25 ± 0.03, 1.00 ± 0.09 vs 0.36 ± 0.04, p < 0.05; Figures 1(c) and (d)). These findings indicated that LncRNA RSU1P2 was up-regulated in liver cancer.

### LncRNA RSU1P2 increased liver cancer cell proliferation, invasion, EMT, and cancer stem cell-related genes expressions

**Figure 2.**
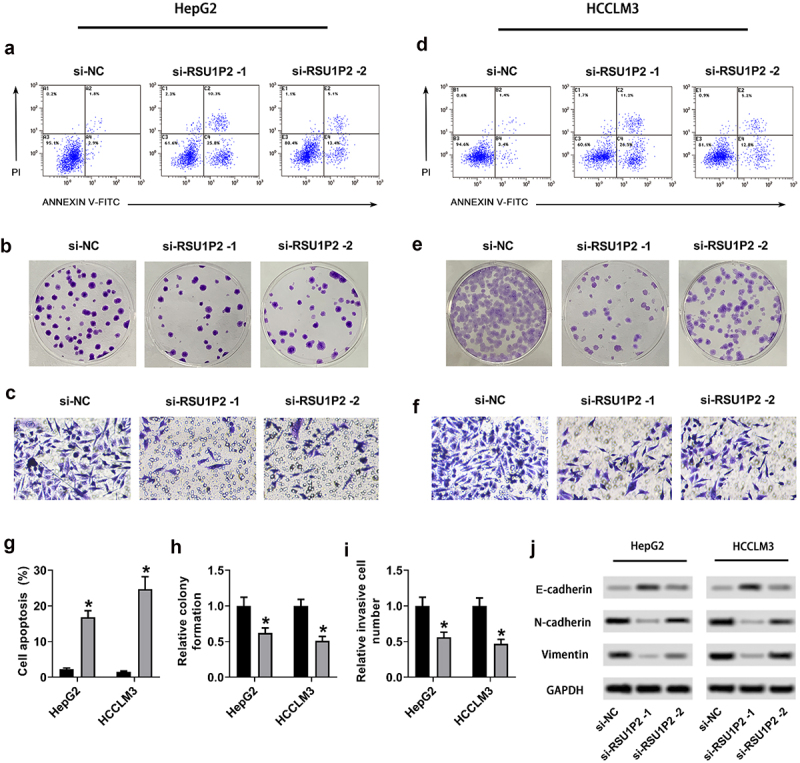
LncRNA RSU1P2 accelerated liver cancer cell proliferation, invasion, EMT and cancer stem cell-related genes expressions. HCCLM3 and HepG2 cells were transfected with si-NC or si-RSU1P2. (a). HepG2 cells apoptosis was examined using flow cytometry. (b). HepG2 cells proliferation was tested using colony formation assay. C. HepG2 cells invasion was tested using Transwell assay. (d). HCCLM3 cells apoptosis was examined using flow cytometry. (e). HCCLM3 cells proliferation was tested using colony formation assay. (f). HCCLM3 cells invasion was tested using Transwell assay. (g). The percentage of HCCLM3 and HepG2 cells apoptosis. (h). Relative colony formation of HCCLM3 and HepG2 cells. (i). Relative invasive cell number of HCCLM3 and HepG2 cells. (j). Expressions of EMT-related proteins E-cadherin, N-cadherin and vimentin were measured by Western blotting. *p < 0.05, compared with si-NC group.

The progression of liver cancer is closely related with the proliferation, cell viability, and invasion of liver cancer cells, so we conduct a series of experiments to measure each of these factors. After knockdown of RSU1P2 in HCCLM3 and HepG2 cells, the apoptosis of HCCLM3 and HepG2 cells was significantly promoted in si-RSU1P2-1 and si-RSU1P2-2 groups compared to si-NC group (HepG2 cells: 4.50 ± 0.48 vs 32.90 ± 4.12%, 4.50 ± 0.48 vs 16.8 ± 2.43%, p < 0.05; HCCLM3 cells: 4.80 ± 0.54 vs 34.70 ± 4.16%, 4.80 ± 0.54 vs 20.50 ± 2.67%, p < 0.05; Figure 2(a,d) and (g)). Colony formation assay showed that HCCLM3 and HepG2 cells proliferation was significantly inhibited in si-RSU1P2-1 and si-RSU1P2-2 groups compared to si-NC group (HepG2 cells: 1.00 ± 0.12 vs 0.43 ± 0.07, 1.00 ± 0.12 vs 0.65 ± 0.08, p < 0.05; HCCLM3 cells: 1.00 ± 0.09 vs 0.40 ± 0.06, 1.00 ± 0.09 vs 0.59 ± 0.07, p < 0.05; Figure 2(b,e) and (h)). The invasion of HCCLM3 and HepG2 cells was significantly inhibited in si-RSU1P2-1 and si-RSU1P2-2 groups than that in si-NC group (HepG2 cells: 1.00 ± 0.12 vs 0.51 ± 0.07, 1.00 ± 0.12 vs 0.61 ± 0.09, p < 0.05; HCCLM3 cells: 1.00 ± 0.11 vs 0.43 ± 0.07, 1.00 ± 0.11 vs 0.57 ± 0.08, p < 0.05; Figure 2(c,f) and (i)). To investigate liver cancer metastasis, EMT proteins (E-cadherin, N-cadherin, vimentin) were detected in HCCLM3 and HepG2 cells. Expressions of EMT-related protein E-cadherin were elevated in si-RSU1P2-1 and si-RSU1P2-2 groups than that in si-NC group, whereas N-cadherin and vimentin were down-regulated in si-RSU1P2-1 and si-RSU1P2-2 group compared to si-NC group (p < 0.05; Figure 2(j) and Supplementary Figure S1B and S1C). Cell viability of HCCLM3 and HepG2 cells was significantly reduced in si-RSU1P2-1 and si-RSU1P2-2 groups than that in si-NC group (HepG2 cells: 1.00 ± 0.11 vs 0.51 ± 0.07, 1.00 ± 0.11 vs 0.67 ± 0.08, p < 0.05; HCCLM3 cells: 1.00 ± 0.12 vs 0.45 ± 0.06, 1.00 ± 0.12 vs 0.64 ± 0.07, p < 0.05; Supplementary Figure S1A). Stem cell marker proteins (ABCG2, NANOG, ALDH1, OCT4, and SOX2) were detected in HCCLM3 and HepG2 cells. Expressions of cancer stem cell-related genes ABCG2, NANOG, ALDH1, OCT4, and SOX2 were significantly down-regulated in si-RSU1P2-1 and si-RSU1P2-2 groups compared to si-NC group (p < 0.05; Supplementary Figure S1D and S1E). These findings suggested that RSU1P2 knockdown repressed liver cancer cell proliferation, invasion, EMT, and the expression of cancer stem cell-related genes.

### Let-7a expression was regulated by LncRNA RSU1P2

**Figure 3.**
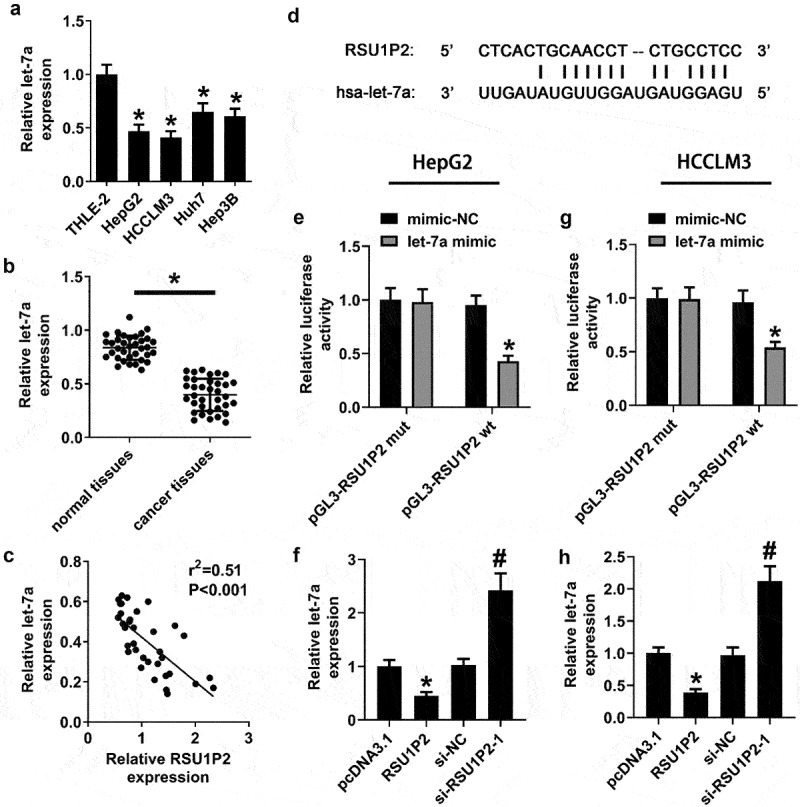
Let-7a expression was regulated by LncRNA RSU1P2. (a). Let-7a expression in normal liver cell line THLE-2 and different liver cancer cell lines was tested using qRT-PCR. *p < 0.05, compared with THLE-2 group. (b). Let-7a expression in normal and cancer tissues was measured by qRT-PCR. *p < 0.05, compared with normal tissue group. (c). Correlation analysis of LncRNA RSU1P2 and let-7a expression. (d). Binding sites between RSU1P2 and let-7a. (e). The regulation of LncRNA RSU1P2 on let-7a in HepG2 cells was detected by dual luciferase reporter gene assay. *p < 0.05, compared with mimic-NC group. (g). The regulation of LncRNA RSU1P2 on let-7a in HCCLM3 cells was detected by dual luciferase reporter gene assay. *p < 0.05, compared with mimic-NC group. (f) and (h). Let-7a expression in HCCLM3 and HepG2 cells transfected with pcDNA3.1-RSU1P2 or si-RSU1P2 was examined using qRT-PCR. *p < 0.05, compared with pcDNA3.1 group; #p < 0.05, compared with si-NC group.

The expression of let-7a was significantly decreased in liver cancer cell lines HepG2, HCCLM3, Huh7, and Hep3B than that in normal liver cell-line THLE-2 (p < 0.05; Figure 3(a)). The expression of let-7a was significantly down-regulated in cancer tissues compared to normal tissues (0.84 ± 0.11 vs 0.39 ± 0.15, p < 0.05; Figure 3(b)). Correlation analysis showed that relative let-7a expression was negatively correlated with LncRNA RSU1P2 expression (p < 0.05; Figure 3(c)). There were binding sites between RSU1P2 and let-7a (Figure 3(d)). In addition, let-7a mimic significantly repressed pGL3-RSU1P2 wt luciferase activity in HCCLM3 and HepG2 cells (HepG2 cells: 1.00 ± 0.11 vs 0.43 ± 0.04; HCCLM3 cells: 1.00 ± 0.09 vs 0.54 ± 0.05, p < 0.05; Figure 3(e) and (g)). Whereas pGL3-RSU1P2 mut luciferase activity was not changed by let-7a mimic in HCCLM3 and HepG2 cells (p > 0.05; Figure 3(e) and (g)). Further transfection experiments showed that RSU1P2 overexpression significantly restrained let-7a expression in HCCLM3 and HepG2 cells, whereas RSU1P2 knockdown significantly up-regulated let-7a expression in HCCLM3 and HepG2 cells (p < 0.05; Figure 3(f) and (h)).

### Let-7a targeted to Tex10

**Figure 4.**
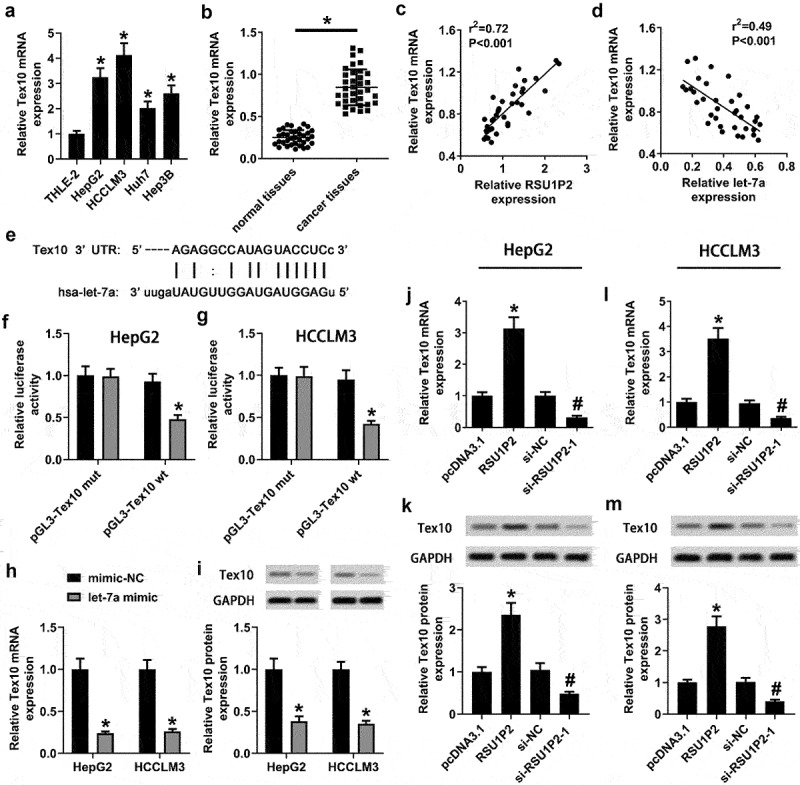
Tex10 expression was regulated by let-7a. (a). Tex10 mRNA expression in normal liver cell line THLE-2 and different liver cancer cell lines was tested using qRT-PCR. *p < 0.05, compared with THLE-2 group. (b). Tex10 mRNA levels in normal and cancer tissues were measured by qRT-PCR. *p < 0.05, compared with normal tissue group. (c). Correlation analysis of LncRNA RSU1P2 and Tex10 expression. (d). Correlation analysis of let-7a and Tex10 expression. E. Binding sites between Tex10 and let-7a. (f-g). The regulation of let-7a on Tex10 in HCCLM3 and HepG2 cells was detected by dual luciferase reporter gene assay. *p < 0.05, compared with mimic-NC group. (h-i). Tex10 mRNA and protein expressions in HCCLM3 and HepG2 cells transfected with mimic-NC or let-7a mimic were measured using qRT-PCR and Western blotting. *p < 0.05, compared with mimic-NC group. (j-m). Tex10 mRNA and protein expressions in HCCLM3 and HepG2 cells transfected with pcDNA3.1-RSU1P2 or si-RSU1P2 were tested using qRT-PCR and Western blotting. *p < 0.05, compared with pcDNA3.1 group; #p < 0.05, compared with si-NC group.

Previous report has found that Tex10 is raised in liver cancer tissues and cells, and Tex10 dysregulation is closely related with cancer stem cell properties [25]. In this study, Tex10 mRNA level was significantly raised in liver cancer cell lines HepG2, HCCLM3, Huh7, and Hep3B than that in normal liver cell-line THLE-2 (p < 0.05; Figure 4(a)). Tex10 mRNA level was significantly elevated in cancer tissues (0.25 ± 0.09 vs 0.85 ± 0.21, p < 0.05; Figure 4(b)). Correlation analysis showed that relative Tex10 mRNA level was negatively correlated with RSU1P2 expression (p < 0.05; Figure 4(c)). Correlation analysis also showed that relative Tex10 mRNA level was negatively related with let-7a expression (p < 0.05; Figure 4(d)). There were binding sites between Tex10 and let-7a (Figure 4(e)). In addition, let-7a mimic significantly suppressed pGL3-Tex10 wt luciferase activity in HCCLM3 and HepG2 cells (HepG2 cells: 1.00 ± 0.11 vs 0.48 ± 0.05; HCCLM3 cells: 1.00 ± 0.10 vs 0.41 ± 0.03, p < 0.05; Figure 4(f) and (g)). Whereas pGL3-Tex10 mut luciferase activity was not significantly changed by let-7a mimic in HCCLM3 and HepG2 cells (p > 0.05; Figure 4(f) and (g)). After transfection of let-7a mimic, Tex10 mRNA, and protein expressions were significantly lowered in HCCLM3 and HepG2 cells (p < 0.05; Figure 4(h) and (i)). RSU1P2 overexpression significantly up-regulated Tex10 mRNA and protein expressions in HCCLM3 and HepG2 cells, whereas RSU1P2 knockdown significantly down-regulated Tex10 mRNA and protein expression in HCCLM3 and HepG2 cells (p < 0.05; Figure 4(j–l) and (m)).

### Let-7a/Tex10 axis was involved in the regulation of RSU1P2 on liver cancer cell proliferation, invasion, EMT, and the expression of cancer stem cell-related genes

**Figure 5.**
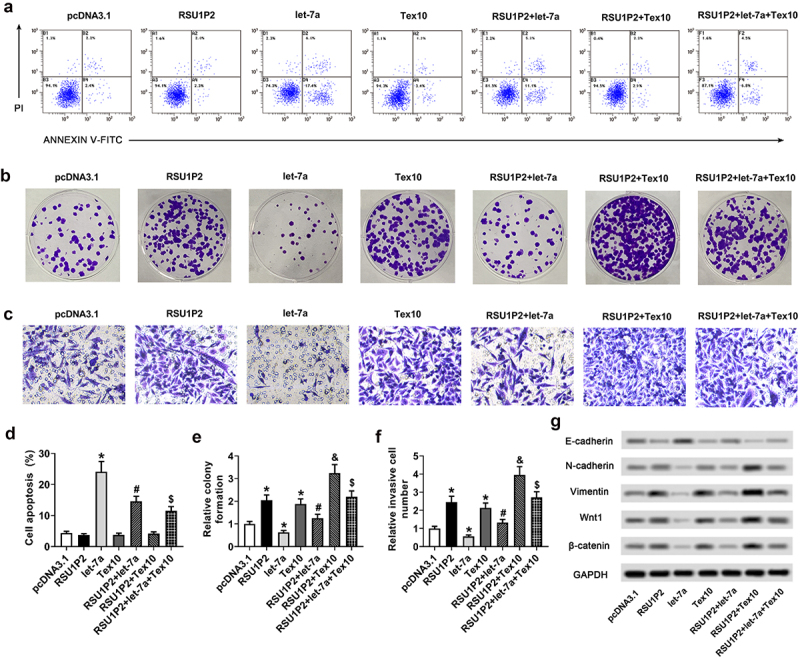
Let-7a/Tex10 axis was involved in the regulation of RSU1P2 on liver cancer cell proliferation, invasion, EMT and the expression of cancer stem cell-related genes. HCCLM3 cells were transfected with pcDNA3.1, pcDNA3.1-RSU1P2, let-7a, pcDNA3.1-Tex10, pcDNA3.1-RSU1P2+ let-7a, pcDNA3.1-RSU1P2+ Tex10. or pcDNA3.1-RSU1P2+ let-7a+Tex10. (a). HCCLM3 cells apoptosis was detected using flow cytometry. (b). HCCLM3 cells proliferation was detected using colony formation assay. (c). HCCLM3 cells invasion was detected using Transwell assay. (d). The percentage of HCCLM3 cells apoptosis. (e). Relative colony formation of HCCLM3 cells. F. Relative invasive cell number of HCCLM3 cells. (g). Expressions of EMT-related proteins E-cadherin, N-cadherin, vimentin, Wnt1 and β-catenin were measured using Western blotting. *p < 0.05, compared with pcDNA3.1 group; #p < 0.05, compared with RSU1P2 group; &p < 0.05, compared with RSU1P2 group; $ p < 0.05, compared with RSU1P2 + let-7a group.

Compared with the RSU1P2 group, cell apoptosis was promoted in the RSU1P2 + let-7a group (3.74 ± 0.39% vs 14.60 ± 1.61%, p < 0.05); the promotion of cell apoptosis by the co-overexpression RSU1P2 and let-7a was abolished in the RSU1P2 + let-7a + Tex10 group (14.60 ± 1.61% vs 11.50 ± 1.36%, p < 0.05; Figure 5(a) and 5D). The cell apoptosis in the pcDNA3.1 group, RSU1P2 group, Tex10 group, and RSU1P2 + Tex10 group showed no obvious differences (Figure 5(a) and (d)). The increased cell proliferation and invasion induced by RSU1P2 upregulation was suppressed in the RSU1P2 + let-7a group but elevated in the RSU1P2 + Tex10 group (cell proliferation: 2.04 ± 0.24 vs 1.25 ± 0.18, 2.04 ± 0.24 vs 3.24 ± 0.38, p < 0.05; cell invasion: 2.45 ± 0.33 vs 1.33 ± 0.17, 2.45 ± 0.33 vs 3.96 ± 0.45, p < 0.05); the suppression of cell proliferation and invasion by the co-overexpression RSU1P2 and let-7a was abolished in the RSU1P2 + let-7a + Tex10 group (cell proliferation: 1.25 ± 0.18 vs 2.20 ± 0.26; cell invasion: 1.33 ± 0.17 vs 2.72 ± 0.31, p < 0.05; Figure 5(b–e) and (f)). Compared with the pcDNA3.1 group, the expressions of N-cadherin, vimentin, Wnt1, and β-catenin were up-regulated by RSU1P2 or Tex10 overexpression, whereas down-regulated by let-7a overexpression; RSU1P2-induced upregulation of N-cadherin, vimentin, Wnt1, and β-catenin were suppressed by let-7a overexpression, but aggravated by Tex10 overexpression; Furthermore, compared with the RSU1P2 + let-7a group, the expressions of N-cadherin, vimentin, Wnt1, and β-catenin were markedly elevated in the RSU1P2 + let-7a + Tex10 group (p < 0.05; Figure 5(g), Supplementary Figure S2C, S2D, and S2E). Compared with the pcDNA3.1 group, the expression of Tex10 was up-regulated by RSU1P2 or Tex10 overexpression (p < 0.05), whereas down-regulated by let-7a overexpression (p < 0.05); RSU1P2-induced upregulation of Tex 10 was suppressed by let-7a overexpression but aggravated by Tex10 overexpression (p < 0.05); Furthermore, compared with the RSU1P2 + let-7a group, the expression of Tex10 was markedly elevated in the RSU1P2 + let-7a + Tex10 group (p < 0.05; Supplementary Figure S2A). The increased cell viability induced by RSU1P2 upregulation was suppressed in the RSU1P2 + let-7a group but further elevated in the RSU1P2 + Tex10 group (p < 0.05); the suppression of cell viability by the co-overexpression RSU1P2 and let-7a was abolished in the RSU1P2 + let-7a + Tex10 group (p < 0.05; Supplementary Figure S2B). Compared with the pcDNA3.1 group, the expressions of cancer stem cell-related genes ABCG2, NANOG, ALDH1, OCT4, and SOX2 were up-regulated by RSU1P2 or Tex10 overexpression, whereas down-regulated by let-7a overexpression; RSU1P2-induced upregulation of the above genes were suppressed by let-7a overexpression but aggravated by Tex10 overexpression; Furthermore, compared with the RSU1P2 + let-7a group, the expressions of ABCG2, NANOG, ALDH1, OCT4, and SOX2 were markedly elevated in the RSU1P2 + let-7a + Tex10 group (p < 0.05; Supplementary Figure S2F).

### *RSU1P2 regulated tumor growth and EMT* in vivo

**Figure 6.**
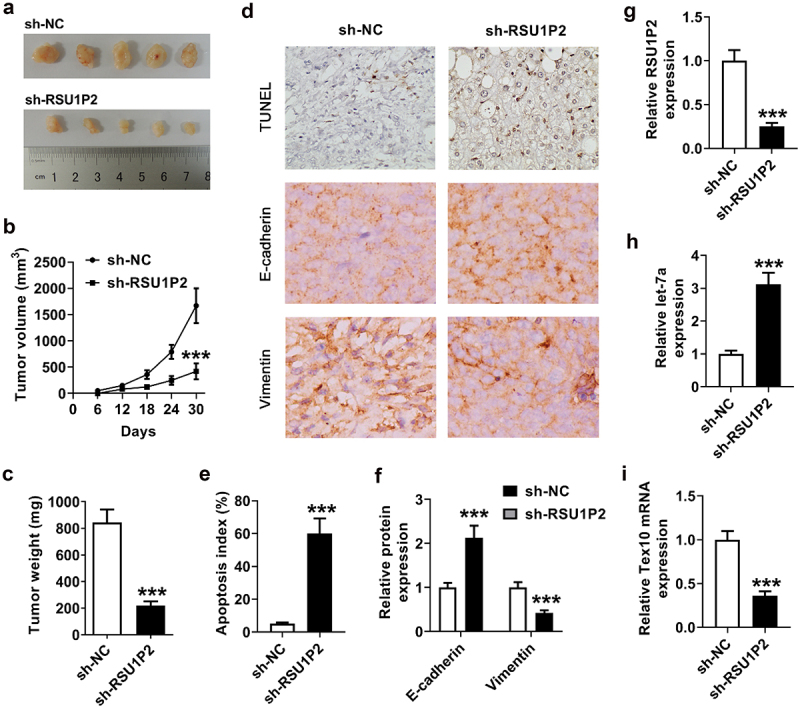
RSU1P2 regulated tumor growth and EMT *in vivo*. HCCLM3 cells transfected with sh-NC or sh-RSU1P2 were subcutaneously injected into nude mice. (a-b). Tumor volume was detected in each group. (c). Tumor weight was detected in each group. (d-f). Expressions of RSU1P2, let-7a and Tex10 were detected by qRT-PCR. G. Apoptosis index (%) was detected in sh-NC and sh-RSU1P2 groups. H. E-cadherin and Vimentin expressions in tumor tissues were examined using IHC staining. ***p < 0.001, compared with sh-NC group.

Tumor volume and weight were significantly reduced in sh-RSU1P2 group (p < 0.001; Figure 6(a,b) and (c)). E-cadherin expression was significantly elevated in tumor tissue of sh-RSU1P2 group, and Vimentin expression was significantly down-regulated in sh-RSU1P2 group (p < 0.001; Figure 6(d) and (f)). In addition, apoptosis index was significantly increased in sh-RSU1P2 group (p < 0.001; Figure 6(d) and (e)). Besides, RSU1P2 expression was significantly inhibited in tumor tissue of sh-RSU1P2 group (1.00 ± 0.12 vs 0.25 ± 0.05, p < 0.001; Figure 6(g)), let-7a expression was significantly raised in tumor tissue of sh-RSU1P2 group (1.00 ± 0.14 vs 3.12 ± 0.35, p < 0.001; Figure 6(h)), and Tex10 mRNA level was significantly repressed in tumor tissue of sh-RSU1P2 group (1.00 ± 0.12 vs 0.35 ± 0.05, p < 0.001; Figure 6(i)).

## Discussion

Cancer-related LncRNAs are usually aberrantly expressed in cancer tissues and cancer cells, which play critical roles in regulating tumorigenesis and cancer stem cell-like properties [38,39]. Various LncRNAs are aberrantly expressed in liver cancer tissues and cells and act as oncogenes or tumor suppressors, thereby affecting multiple biological processes [40,41]. Although some LncRNAs are well identified in liver cancer, it is valuable to clarify potential meaningful LncRNAs in modulating the tumorigenesis and cancer stem cell-like properties in liver cancer [42,43]. In this study, LncRNA RSU1P2 was raised in cancer tissues from liver cancer patients and liver cancer cell lines. RSU1P2 knockdown remarkably decreased RSU1P2 expression in HepG2 and HCCLM3 cells. Thus, we first revealed that LncRNA RSU1P2 was elevated in liver cancer, which suggested that the up-regulation of LncRNA RSU1P2 might exert important function in the progression of liver cancer.

Previous researches have shown that abnormally expressed LncRNAs are vital regulators in controlling the proliferation, invasion, EMT, and cancer stem cell-like properties in liver cancer [44–47]. For example, LncRNA CRNDE was increased in liver cancer tissues and liver cancer cells, and knockdown of CRNDE restrained growth, metastasis of liver cancer tumors, suppressed liver cancer cell EMT and Wnt/β-catenin pathway [48]. LncRNA DANCR was raised in liver cancer tissues and liver cancer cells, and DANCR overexpression facilitated the proliferation and metastasis of liver cancer cells [49]. LncRNA AGAP2-AS1 was increased in liver cancer tissues and liver cancer cells, and AGAP2-AS1 overexpression facilitated cell proliferation, invasion, and migration, EMT of liver cancer [50]. In this study, RSU1P2 knockdown remarkably reduced the cell viability, suppressed the colony formation of liver cancer cells, decreased invasive cells number, inhibited expressions of cancer stem cell-related genes and Vimentin protein expression, increased the apoptosis of liver cancer cells and up-regulated E-cadherin protein expression *in vitro*. These findings indicated that LncRNA RSU1P2 functioned as a cancer-promoting gene in liver cancer. Until now, no other studies have verified the role of LncRNA RSU1P2 in controlling tumorigenesis and cancer stem cell-like properties in liver cancer, which will provide theoretical basis for liver cancer research.

Mounting evidences have provided evidences that LncRNAs play their roles in liver cancer via acting as a ceRNA to sponge miRNAs [51–54]. In this study, RSU1P2 expression was elevated whereas let-7a was remarkably restrained in liver cancer tissues and cells, and let-7a level was negatively related with RSU1P2 expression in liver cancer tissues. Moreover, it was identified that let-7a was a target of RSU1P2 in HCCLM3 and HepG2 cells. Since let-7a suppress the proliferation of liver cancer cells, EMT, renewal of cancer stem cells and Wnt-β-catenin pathway in liver cancer [18]. We therefore considered that LncRNA RSU1P2 acted as an oncogene in liver cancer by negatively regulating let-7a.

Online bioinformatics prediction tools are meaningful in helping researchers clarify the underlying molecular mechanism within miRNAs and their downstream targets [55]. Previous reports have shown that let-7a has binding sites with its downstream molecules and can target to 3’ UTRs of these molecules, such as BCL2L1, STAT3 and RTKN [56–58]. According to the prediction of StarBase (http://starbase.sysu.edu.cn/), there were binding sites between let-7a and 3’ UTRs of Tex10 mRNA. So, we assumed that Tex10 might be a target of let-7a. In this study, inverse correlation between let-7a and Tex10 level was identified in liver cancer tissues. In addition, let-7a overexpression prominently down-regulated Tex10 expression in HCCLM3 and HepG2 cells. Luciferase reporter assay identified that let-7a targeted 3’ UTR of Tex10 mRNA. Therefore, we first confirmed that let-7a acted as the upstream molecule to regulate Tex10 by binding to 3’ UTRs of Tex10, and the methods used in this study were consistent with previous reports [59,60]. Moreover, there was a positive correlation between RSU1P2 and Tex10 in liver cancer tissues, and RSU1P2 positively regulated Tex10 mRNA and protein expressions in HCCLM3 and HepG2 cells. Therefore, we assumed that LncRNA RSU1P2 acted as an oncogene in liver cancer by promoting Tex10 via sponging let-7a.

According to the findings of previous report, highly expressed Tex10 in liver cancer cells and liver cancer stem cells can promote cancer stem cell properties [25]. Although the ability of Tex10 to promote self-renewal of stem cells have been confirmed, Tex10 is still a pluripotency factor that lacks concern and in-depth understanding [23,24]. Thus, the underlying mechanism of Tex10 in liver cancer is needed to be clarified. In this study, Tex10 restoration abolished the inhibition effect of RSU1P2+ let-7a on cell viability, proliferation, invasion, EMT of liver cancer cells, and the expressions of cancer stem cell-related genes. Therefore, our findings suggested that Tex10 was involved in LncRNA RSU1P2/let-7a mediated promotion of the tumorigenesis and cancer stem cell-like properties in liver cancer. Thus, we have revealed the role of LncRNA RSU1P2/let-7a/Tex10 pathway in controlling tumorigenesis and cancer stem cell-like properties in liver cancer, which may supply potential therapeutic targets for liver cancer treatment.

Wnt/β-catenin pathway, an important driver of tissue stem cells in mammals, can promote cancer progression and metastasis through controlling the growth and biology of stem cells [61]. In liver cancer treatment, targeting Wnt/β-catenin pathway components can achieve anticancer effect by reducing hepatocarcinogenesis and the maintenance of liver cancer stem cells [62]. LncRNAs/miRNAs are important modulators in regulating cancer stemness and cancer cell proliferation by enhancing Wnt/β-catenin pathway [63,64]. Xiang et al have shown that highly expressed Tex10 facilitates EMT and stemness of ESCC cells through activating Wnt/β-catenin pathway [65], indicating Tex10 might exert its promotion function in cancers through Wnt/β-catenin pathway. In this study, RSU1P2 facilitated Wnt1 and β-catenin expressions, RSU1P2+ let-7a down-regulated Wnt1 and β-catenin expressions, and RSU1P2+ let-7a+Tex10 abolished the inhibition effect on Wnt1 and β-catenin protein expressions, indicating RSU1P2/let-7a/Tex10 regulated Wnt/β-catenin pathway in liver cancer.

## Conclusion

LncRNA RSU1P2 functions as a cancer-promoting gene in liver cancer and promotes tumorigenesis *in vivo*. Besides, LncRNA RSU1P2 facilitates liver cancer cells proliferation, invasion, EMT, and cancer stem cell-like properties through let-7a/Tex10 pathway *in vitro*.
